# The Effect of Herbal Supplements on Blood Pressure: Systematic Review and Meta-Analysis

**DOI:** 10.3390/antiox11081419

**Published:** 2022-07-22

**Authors:** Anna Lipert, Iwona Szadkowska, Ewelina Matusiak-Wieczorek, Ewa Kochan

**Affiliations:** 1 Department of Sports Medicine, Medical University of Lodz, 92-213 Lodz, Poland; iwona.szadkowska@umed.lodz.pl (I.S.); ewelina.matusiak-wieczorek@umed.lodz.pl (E.M.-W.); 2 Department of Pharmacological Biotechnology, Medical University of Lodz, 90-152 Lodz, Poland; ewa.kochan@umed.lodz.pl

**Keywords:** hypertension, plant supplements, beetroot juice, cherry juice, barberry, bergamot, resveratrol, pycnogenol

## Abstract

Herbal supplements rich in phenolic compounds are evidenced to have a protective effect against cardiovascular diseases. Therefore, they are suggested to be included in diets for people with hypertension (HT). HT is a global health problem and is estimated to affect billions of people until the end of 2025. For this reason, every possible and effective solution preventing HT should be considered. The aim was to perform an updated meta-analysis and review of recently published studies to evaluate the effect of selected herbal supplements on blood pressure reduction. We searched the PubMed database with specified selection criteria, analysing the RCT studies from 2011 to 2021. A total of 31 studies were included in the analysis, and the meta-analysis was conducted on the data from 16 of them. The general effect size of all the supplements via placebo was d = 1.45, *p* < 0.05 for systolic blood pressure (SBP) and d = 0.31, *p* < 0.05 for diastolic blood pressure (DBP). The meta-analysis and review of the literature demonstrated that herbal supplements, such as resveratrol, cherry juice, beetroot juice, bergamot extracts, barberry, and pycnogenol, can be effective in blood pressure reduction and cardiovascular prevention, but attention should be paid to their appropriate dosage due to the possibility of side effects from the digestive system.

## 1. Introduction

According to the recommendations of the European Society of Cardiology (ESC) and the European Society of Hypertension (ESH), hypertension (HT) is diagnosed if the systolic blood pressure (SBP) is 140 mmHg and over or if the diastolic blood pressure (DBP) is 90 mmHg and over [1]. The recommendations of the American College of Cardiology (ACC) and American Heart Association (AHA) take more restrictive values of blood pressure (BP) for the diagnosis of HT. ACC/AHA recommendations define stage 1 hypertension as SBP 130–139 mmHg or DBP 80–89 mmHg, while the European guidelines classify this as high normal BP (130–139/85–89 mmHg) [2].

Although either an elevated systolic or an elevated diastolic blood pressure are the criteria for the diagnosis of HT, more attention is given to SBP as a major risk factor for the increased incidence of cardiac and vascular diseases for people aged 50 and over [3]. According to the current data, every 20-mmHg systolic or 10-mmHg diastolic increase doubles the risk of death from ischemic heart disease and stroke among people aged 40 and over [3,4]. Hypertension [5] is a global health problem affecting about 1 billion people [6], and it is estimated that up to year 2025 it will reach the number of 1.5 billion [7].

There are various recommended strategies to prevent HT, including lifestyle modifications by changing diet and undertaking physical activity or drug interventions [8]. The modifications in diet concern increases in the consumption of fresh fruits and vegetables and low-fat dairy products and the reduction of sodium [9,10]. In addition, the use of nutraceuticals supplemented in everyday diets, which can lower BP and may delay or circumvent drug therapy, has received much attention in the literature [8,11,12]. Products recommended in cardiovascular disease prevention are those rich in phenolic compounds that are vital in anti-aging, anti-inflammatory, antioxidant, and anti-proliferative activities [13,14].

Naturally, phenolics occur in foods such as grapes, wine, grape juice, berries, or pomegranate juice; however, their content strongly depends on cultivation, technology processes, and transformation [15].

Numerous research studies present the effectiveness of the diet alone or in combination with other lifestyle changes in BP decrease across a wide range of BP levels [16] and in the reduction of the risk of other certain chronic diseases [17,18,19]. One of the dietary interventions recognized to be effective in blood pressure reduction is the Dietary Approaches to Stop Hypertension (DASH) diet [20], especially in individuals with high sodium intake [21]. There are also several complementary and alternative therapies that include dietary supplements, functional foods, and traditional herbs for treating hypertension [22,23]. The studies confirmed that the positive impact on the level of BP occurs while supplementing potassium [24], chromium [25], or inorganic nitrate products [16]. In the case of the latter, positive effects on BP were usually observed in short-term trials in clinical settings [26].

Although there are many studies analysing the influence of separate phenol-rich products in relation to their protective effects on cardiovascular protective features [27,28], the literature comprehensively presenting the effects of those supplements on BP is sparce. Therefore, the aim of the study was to systematically evaluate the literature from existing randomised controlled trials on phenolic compounds to help quantify the overall effect of them on blood pressure in adults.

## 2. Materials and Methods

### 2.1. Data Source and Searching Strategy

**Figure 1:**
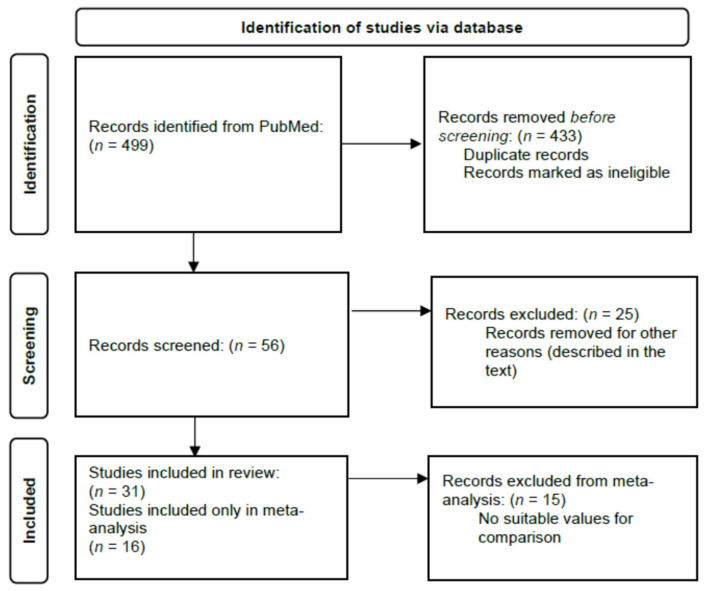
PRISMA flow diagram of the study selection process for all of the articles.

There was undertaken a systematic review and meta-analysis assessing the potential effect of the selected phenolic-rich products on blood pressure response in adults. The inclusion criteria were as follows: (a) natural plant origin compounds; (b) potentially useful in the fight against high blood pressure. All English-language medical literature, with the time restriction from 2011 to 2021, was searched using the electronic PubMed database. The searching strategy was based on the use of various combinations of keywords with the formula: name of substance + key words (Table S1 as Supplementary Material), including only the randomised clinical trials (RCTs) in apparently healthy humans or those with chronic diseases such as hypertension, hypercholesterolemia, type 2 diabetes, obesity, metabolic chronic diseases, atherosclerosis, and heart failure. The articles were excluded because of the following reasons: lack of reference to blood pressure values; lack of supplement level or its administration was together with other supplements; research on cerebrovascular function and cognitive performance; in vitro or animal studies; published only study protocol; studies on patients with pulmonary hypertension, kidney diseases, gynecological diseases, and menopause; access only to the abstract; the articles included non-analysed supplement data. In addition, the exclusion criterion was if research on the supplement discussed its role as (a) an enhancer of endothelial function in response to acute aerobic exercise; (b) a factor regulating gene expression, enzyme activity, oxygen kinetics, and angiogenesis process; (c) a factor reducing pain; (d) a tumor necrosis factor (TNF) inhibitor. Figure 1 illustrates a PRISMA flow diagram of the study selection process for all of the articles. The study followed the guidelines of the Preferred Reporting Items for Systematic Reviews and Meta-analyses (PRISMA) protocol for conducting systematic reviews and meta-analyses normatively [29]. At the time of the protocol submission, the PROSPERO platform was prioritising the registration of COVID-19 protocols, so it was excluded from the registration. To avoid further delay, the current submission passed a basic automated check and was published automatically.

The selected articles had to relate to one of six supplements, such as: resveratrol, pycnogenol, beetroot juice, barberry, bergamot, or cherry juice.

*Resveratrol* is a poly-phenol, natural stilbene phytoalexin (3, 5, 4′-trihydroxystilbene) synthesized, e.g., in leather grapes, in response to pathogen infections, oxidative stress, and UV radiation. Due to the similarities in chemical structure for synthetic oestrogen (diethylstilbestrol), resveratrol is classified as a phytoestrogen, with plant hormone oestrogen-like action [30]. It occurs in both the cis- and trans-isomer form. In high concentrations, it is also present in wine, mainly red. This compound has a wide range of biological activities, e.g., anti-inflammatory, anti-angiogenic, antioxidant, and anti-cancer. In addition, it protects against cardiovascular and neurodegenerative diseases and inhibits LDL cholesterol oxidation, platelet aggregation, and angiogenesis [31].

*Pycnogenol* is a patented product rich in condensed flavonoids extracted from the bark of the French maritime pine tree. It has been shown that pycnogenol has antioxidative and anti-inflammatory activity and inhibits platelet aggregation. There are studies indicating the beneficial effects of pycnogenol supplementation, such as the decrease of blood glucose levels, blood pressure, and the level of urinary albumin [32].

*Beetroot juice (BRJ)* is a highly-concentrated source of inorganic nitrates in the diet. After ingestion, nitrate (NO^3−^) is metabolized to bioactive nitrite (NO^2−^) and then converted into nitric oxide (NO) [33,34]. In the human body, NO is synthesized in various cells via three isoforms of nitric oxide synthase (NOS)—neuronal (nNOS), inducible (iNOS) and endothelial (eNOS). The role of eNOS is the local production of NO as a factor having vasodilatory properties [35]. NO is a principal determinant of normal endothelial and vascular function. In states of inflammation, NO production by the vasculature increases considerably and contributes to oxidative stress [36]. Studies show that the BRJ intake increases NO availability independently on endothelial NO synthesis [35]. The clinical effects of NO include blood pressure reduction and the improvement of blood flow in tissues and organs, which is an important mechanism in the prevention and treatment of cardiovascular diseases [37,38].

*Barberry* is a plant that grows in Asia and Europe. Its fruits, roots, leaves, and flowers are used for medicinal purposes. The traditional use of barberry in folk medicine was detailed in the study of Imenshahidi and Hosseinzadeh [39]. The barberry fruit contains berberine and berberrubine, which have antioxidant properties. What is more, the supplementation of barberry may have a positive effect on the immune system, glucose metabolism, and lipid profiles [40].

*Bergamot* is a plant growing in Southern Italy. It contains high levels of flavonoids. Bergamot has an anti-inflammatory potential, has a strong effect on lipid metabolism, insulin sensitivity, and glucose tolerance, and it can reduce the sensation of hunger. Bergamot has a positive influence on cardiovascular health, acting as a protector against free radical damage, for example, in the vascular endothelium [41,42].

*Cherry juice* is a rich source of antioxidants and flavonoids. The supplementation of cherry juice has a beneficial anti-inflammatory effect. What is more, there are also studies indicating that cherry juice can lower systolic BP and LDL cholesterol; thus, it also has a cardioprotective effect [43].

The initial screening phase based on the titles and abstracts was provided to exclude the irrelevant records, animal or in vitro studies, and conference papers to obtain only those relevant, irrespective of where they were conducted and whether they are full text. In addition, the duplicate articles were found and excluded. To assess study eligibility, three co-authors were involved in the literature search, and one of the co-authors was an independent reviewer of the records for inclusion and exclusion criteria of the relevant articles. The primary outcomes of the analyses were mean differences of both SBP and DBP between intervention and control groups; secondary outcomes were mean changes in SBP and DBP between the baseline and final of the intervention or placebo group. Exclusion reasons for most of these studies were that they were conducted on animals or concerned with other diseases, e.g., kidney disease, autism spectrum, rheumatoid arthritis, sleep problems, skin problems, memory and cognitions problems, breast disease, rheumatoid arthritis, visual problems, and haemorrhoids.

The search for RCTs concerning “**resveratrol**” resulted in 158 articles, including 51 articles combined with “cardiovascular disease”, 32 with “blood pressure”, 10 with “hypertension”, 26 with “endothelial function” (including FMD-flow-mediated dilation or/and PWV pulse wave velocity), 35 with “vascular function”, and 4 with “arterial stiffness”. After the full text analysis, there were 92 articles excluded. There were 48 articles duplicated. The final number of articles for screening was 18. Some articles were excluded after the screening phase because the presentation of the data did not allow for a comparison with other papers. The final number of articles was 5.

The search for RCTs concerning “**pycnogenol**” resulted in 26 articles, including 6 articles combined with “cardiovascular disease”, 4 with “blood pressure”, 1 with “hypertension”, 23 with “endothelial function” (including FMD—flow-mediated dilation—or/and PWV pulse wave velocity), 46 with “vascular function”, and 9 with “arterial stiffness”. After the full text analysis, there were 21 articles excluded. There were 8 articles duplicated. The final number of articles for screening was 3.

The search for RCTs concerning “**beetroot juice**” resulted in 248 articles, including 49 articles combined with “cardiovascular disease”, 99 with “blood pressure”, 22 with “arterial hypertension” (except pulmonary HT), 4 with “endothelial function” (including FMD—flow-mediated dilation—or/and PWV pulse wave velocity), 8 with “vascular function”, and 3 with “arterial stiffness”. After the full text analysis, there were 220 articles excluded. There were 135 articles duplicated. The final number of articles for screening was 28. Some articles were excluded after the screening phase because the presentation of the data did not allow for a comparison with other papers. The final number of articles was 16.

The search for RCTs concerning “**barberry**” resulted in 14 articles, including 7 articles combined with “cardiovascular disease”, 3 with “blood pressure”, 2 with “arterial hypertension” (except pulmonary HT), 1 with “endothelial function” (including FMD—flow-mediated dilation—or/and PWV pulse wave velocity), 1 with “vascular function”, and 0 with “arterial stiffness”. After the full text analysis, there were 13 articles excluded. There were 3 articles duplicated. The final number of articles was 1.

The search for RCTs concerning “**bergamot**” resulted in 6 articles, including 2 articles combined with “cardiovascular disease”, 2 with “blood pressure”, 1 with “arterial hypertension” (except pulmonary HT), 0 with “endothelial function” (including FMD—flow-mediated dilation—or/and PWV pulse wave velocity), 1 with “vascular function”, and 0 with “arterial stiffness”. After the full text analysis, there were 4 articles excluded. There were 0 articles duplicated. The final number of articles was 2.

The search for RCTs concerning “**cherry juice**” resulted in 47 articles, including 10 articles combined with “cardiovascular disease”, 14 with “blood pressure”, 6 with “arterial hypertension” (except pulmonary HT), 2 with “endothelial function” (including FMD—flow-mediated dilation—or/and PWV pulse wave velocity), 7 with “vascular function”, and 4 with “arterial stiffness”. After the full text analysis, there were 43 articles excluded. There were 22 articles duplicated. The final number of articles was 4.

### 2.2. Statistical Analysis

Statistical analyses were conducted with the use of StatSoft software (version StatSoft version 1.0.67. www.statsoft.pl, accessed on 10 June 2022). The standardized effect size and 95% confidence intervals (95% CI) were calculated to weigh the results of the included studies. Considering acceptable heterogeneity status or homogeneity, the random-effect meta-regression model was used. The statistical significance was considered if *p* < 0.05.

## 3. Results

### 3.1. Analysis of the Supplements SBP Effectiveness

**Figure 2:**
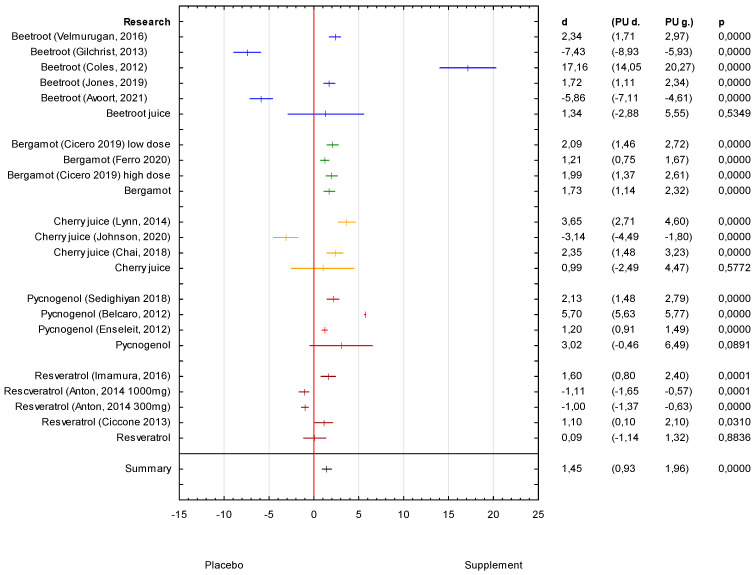
Forest plot of the results of the meta-analysis for supplement effectiveness on SBP versus the placebo group. Data are shown as mean effect sizes with 95% CI.

The analysis of the collected results from the randomised controlled trials (RCTs) showed a positive effect of the supplements on SBP (d = 1.45 (95% CI 0.93 to 1.96)), with *p* < 0.05 (Figure 2). The strongest effect on SBP was observed in the research on pycnogenol (d = 3.02 (95% CI −0.46 to 6.49)), which was administered for at least 2 months (Table 1). The strongest effect statistically significant was showed in the Belcaro 2012 study, with *p* < 0.001 (Figure 2). The next supplements that were noticed to influence SBP were bergamot (d = 1.73 (95% CI 1.14 to 2.32)), with *p* < 0.001, and beetroot juice (d = 1.34 (95% CI −2.88 to 5.55)) (Figure 2). All of the research on bergamot showed a positive effect on SBP (Figure 2). However, the results from the research on beetroot juice were not so clear. Three of five studies demonstrated the clinically important effects for SBP decrease (Coles 2012; Velmurugan 2016 and Jones 2019), and the last two showed no effect (Figure 2). Those two studies (Gilchrist 2013; Avoort 2021) with no effect to the placebo differed in the age of the study sample, which was over 65 years or the time of administration, lasting only 2 weeks (Table 1). The research on beetroot juice, shown in Table 2, also suggested that the clinical effect of the supplement can be obtained if the dosage is higher. The smallest effect on SBP was observed in the research on resveratrol (d = 0.09 (95% CI −1.14 to 1.32)) (Figure 2). The results of two studies showed a positive effect (Imamura 2016; Ciccone 2013), but they were characterized with a high dosage of the supplement or a longer time of administration in comparison to the study with no effect on SBP (Table 1). It was also observed based on the results from the research on resveratrol demonstrated in Table 2. There was also one study demonstrating the effect of barberry juice on SBP decrease (Table 2).

### 3.2. Analysis of the Supplements DBP Effectiveness

**Figure 3:**
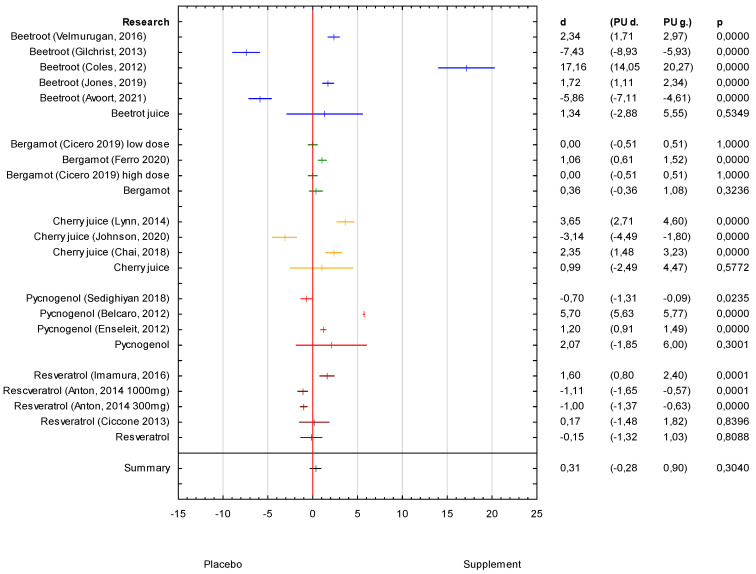
Forest plot of the results of the meta-analysis for supplement effectiveness on DBP versus the placebo group. Data are shown as mean effect sizes with 95% CI.

In general, the analysis of the collected results showed a positive but rather moderate effect of the supplements on DBP (d = 0.31 (95% CI −0.28 to 0.90)) with no statistical significance (Figure 3). The strongest effect on DBP was also observed in the research on pycnogenol (d = 2.07 (95% CI −1.85 to 6.00)), but the results were less unambiguous in comparison to the results on SBP (Table 1). The strongest effect that was statistically significant was shown in the Belcaro 2012 study with *p* < 0.001. There was one study in which the results did not show the effect on DBP (Sedighiyan 2018), but it may be dependent on the time of supplement administration, which may be necessary to be longer at a dose of 150 mg to have a reducing effect on DBP (Table 2). The next supplement that was noticed to influence DBP was beetroot juice (d = 1.34 (95% CI −2.88 to 5.55)) (Figure 2). The results from three of five studies (Velmurugan 2016; Coles 2012; Jones 2019) demonstrated the clinically important effects of DBP decrease and what was similar to SBP (Figure 3). The results of two studies showed no effect that could be influenced by the participants age or the dosage of the administered supplement (Figure 3, Table 1 and Table 2). The smallest influence on DBP was observed in research on bergamot (d = 0.36 (95% CI −0.36 to 1.08)), although some clinical effect on SBP decrease was observed (Figure 2 and Figure 3). Ferro (2020) showed the strongest effect of bergamot (d = 1.06 (95% CI 0.61 to 1.52)), which was statistically significant with *p* < 0.001. The study with no effect on the DBP decrease was in the study with a shorter time of administration, which may result in different DBP improvements (Table 1). The negative effect on DBP was observed in the research on resveratrol (Figure 3 and Table 1). Only one of four studies (Imamura 2016) showed a strong positive effect of resveratrol on DBP (d = 1.60 (95% CI 0.80 to 2.40) with *p* < 0.05, but it was a study with a long-term follow-up (Figure 3 and Table 1). The results of the research described in Table 2 also suggest that the effectiveness on DBP may be depended on time or dosage. It was also observed that the study participants involved into the study were also those over 65 years, which can also influence the results (Table 2). The results of research on resveratrol that showed the positive effect on BP were usually people aged below 65 years (Table 1 and Table 2). In addition, the barberry juice can influence DBP, which was observed in one study (Table 2).

## 4. Discussion

The present study is a comprehensive systematic review and meta-analysis of several dietary supplements, such as pycnogenol, resveratrol, beetroot juice, barberry, bergamot, or cherry juice, recognized to be effective in blood pressure (BP) decrease, including 31 RCTs (1250 participants). Overall, the results demonstrated that most of them can be clinically effective in decreasing BP and improving cardiovascular function in different patients; however, the supplement effectiveness may be dependent on the dosage of the bioactive compound or the time of administration. To our knowledge, this is the first this kind of paper gathering information on several supplements that are recommended in the prevention of cardiovascular diseases.

Based on the results of the present review, it seems that, among different supplements recommended in blood pressure improvement, the supplementation of pycnogenol can bring the most benefits in the prevention of blood pressure. It is in line with a previous review that indicated that pycnogenol supplementation reduced systolic and diastolic blood pressure in trials with more than an 8-week intervention [44]. Pourmasoumi et al. also suggested that a favourable effect on DBP was observed in studies with over a 12-week in duration [45]. Fogacci et al. stated that pycnogenol consumption did not reduce BP levels [46], but the duration across the considered clinical studies varied substantially; thus, the results from long-term trials are required to confirm those findings.

Additional supplements that may be beneficial in the prevention of elevated blood pressure are beetroot juice, bergamot, and cherry juice; however, the effect is not the same on systolic and diastolic BP. Beetroot juice, cherry juice, and bergamot were observed to be the most effective in SBP response; however, the favourable effect on DBP was not so clear. Generally, the positive effect of nitrate supplementation on BP was noticed after medium-term dietary intake in clinical settings [26]. In addition, the previous review comparing the effects of the chronic consumption of cherry juice on the risk of cardiovascular disease confirmed that its supplementation might contribute to an improvement in BP [47]. However, the investigation of the hypotensive properties of beetroot juice also highlighted that no significant change in DBP was observed to its baseline value after beetroot juice treatment [48]. The findings of the present review indicated that the research on beetroot juice with no effect on BP was characterized by smaller doses of the administered supplement or the participants were over 65 years old. The duration of all the studies was at least 2 weeks, which was the shortest time to obtain the positive effect of the supplement on BP [48]. Although it is evidenced that a dose of 500 mL/d of beetroot juice was noticed to have the strongest relationship with blood pressures decrease [48], the study of Velmurugan showed that even 250 mL of nitrate-rich beetroot juice in a 6-week duration can be effective [49]. Therefore, it can be assumed that the age of the individual may have also affect the BP improvement after beetroot juice supplementation. There is little research analysing the different protocols of bergamot in blood pressure response. Nevertheless, the results of the present review suggest that the right dosage of the supplement may influence the DBP reduction.

A single study included in the present review showed some positive effects of barberry on BP in people with type 2 diabetes [40]. Nevertheless, the studies regarding the effect of barberry on cardiovascular risk factor are limited. Recently, Hadi et al. showed that the consumption of barberry can be a safe strategy for the management of lipid parameters [50].

Resveratrol provided the least clinically important effect on blood pressure, both in SBP and DBP reduction, and the most clinically significant effects were observed only in research with a long-term follow-up or a high dosage administration. Fogacci et al. noticed that the favourable effect of resveratrol in order to prevent cardiovascular diseases is mostly when used in a daily dose of ≥300 mg/day [32], especially in people with diabetes. Resveratrol was also observed to be useful in metabolic parameter reduction and HDL increase, but still the effect depended on the high dosage (over 500 mg) or long-term intervention (over 10 weeks) [51]. In contrast, in people with a metabolic syndrome, no significant effect was observed in SBP and DBP reduction [52]. The administration of 3 g of resveratrol daily may be related with experiencing gastrointestinal syndromes [53], which is worth considering when planning therapy with this supplement.

There are several strengths of the present review. Firstly, it was prepared to be in line with the current PRISMA recommendations [54]. Secondly, the review included several supplements that had very similar chemical components or bioactive features considered to be effective in blood pressure response, while other previous reviews were mainly focused only on a particular supplement [45,48,55]. Next, only the single supplement results of RCTs were analysed, as those provided the best evidence on the efficacy of specific pharmacological treatments [56]. Finally, the review included supplements that have been rarely described in previous reviews with meta-analysis [47].

However, some limitations have to be emphasized. The number of studies included in a meta-analysis was relatively small for most supplements. Unfortunately, some of the results from the selected research were presented only graphically in the figures, without giving the average values that were required for the meta-analysis. In the case of extracts, it would be advisable to attach a quantitative and qualitative specification of the composition to verify that the extracts used in different studies did not differ, which could affect the obtained results. In addition, the research process was limited to articles in English, which could introduce bias. The methodological approach of a meta-analysis was to compare each of the supplements with placebo; therefore, a further network meta-analysis might be needed to provide a single, coherent ranking of supplements by performing direct and indirect comparisons to determine which is the optimal supplement.

To sum up, the overall analysis including all the trials showed that the analysed supplements provided clinical meaningful effects on systolic and diastolic blood pressure responses compared with the placebo, mainly in healthy adults or people below 65 years. Further placebo-controlled trials with a higher dosage or a longer treatment duration are required to clearly confirm the efficacy of supplements such as beetroot juice, bergamot, and resveratrol. It would also suggest to further evaluate the safety of using these supplements in longer administrations. The findings from the present review and meta-analysis provided thorough and valuable information for physicians, dietitians, or other health promotion professionals to choose suitable supplements as a cost-effective nutritional approach in the early prevention of elevated blood pressure and cardiovascular outcomes.

## 5. Conclusions

Supplements studied in this paper can be effective in blood pressure reduction and cardiovascular prevention. The best antihypertensive effect was shown by resveratrol and pycnogenol; however, it depended on the dose and frequency of administration. Beetroot juice, cherry juice, and bergamot mainly influenced systolic blood pressure. In the case of using barberry juice, it is not possible to unequivocally determine its influence on blood pressure due to the limited number of publications. To obtain an appropriate clinical effect, it is necessary to define a separate protocol of supplementation for each of them. Further research allowing the establishment of a safe dosage level that does not disturb the digestive system is recommended.

## Figures and Tables

**Table 1:** All studies included in the meta-analysis (*n* = 16).

Name of the Supplement	Author Year	Sample	Supplement Administration Protocol	Duration	Form of Supplement	Significant Results
**Resveratrol**	Anton, 2014	32 overweight adults over 65 y	300 mg/day or 1000 mg/day or placebo	1 month	Product was provided by Reserveage Organics and contained naturally-derived resveratrol from grapes and wild Japanese knotweed (*Polygonum cuspidatum*). Microcrystalline cellulose was used for the placebo.	Daily resveratrol supplementation is generally well-tolerated in overweight, older adults.
	Imamura, 2016	50 patients with T2DM	100 mg/day or placebo	12 weeks	Resveratrol was obtained from the BHN Corporation (Tokyo) as resveratrol-ε, which is derived from grape stems. Supplement of a 100-mg resveratrol (total resveratrol: oligo-stilbene 27.97 mg/100 mg/day) or placebo tablet.	Resveratrol supplementation decreased: systolic BP (−5.5 mmHg), d-ROMs (−25.6 ± 41.8 U.CARR), CAVI (−0.4 ± 0.7), BW (−0.8 ± 2.1 kg,), and body mass index (−0.5 kg/m^2^). Resveratrol supplementation improved arterial stiffness and reduced oxidative stress in patients with T2DM.
	Ciccone, 2013	50 eligible healthy volunteers aged 20–50 years	500 mg/day or placebo	1 month	Supplement Transmax (resveratrol, 500 mg, Biotivia Bioceuticals LLC).	Resveratrol supplementation significantly reduced triglyceride and C-reactive protein (CRP) concentrations and increased Total Antioxidant Status (TAS) values.
**Cherry juice**	Chai, 2018	37 healthy adults, aged 65–80 years	480 mL of tart cherry juice or control drink per day	12 weeks	A total of 68 mL of Montmorency tart cherry concentrate (King Orchards, MI, USA) was diluted with 412 mL water. The control drink had the colour and taste as close as possible to the tart cherry juice, and it was prepared by mixing unsweetened black cherry-flavoured Kool-Aid (Kraft Foods, United States) with water. Drinks were consumed twice a day (in the morning and evening) in a dose of 240 mL per time.	Tart cherry juice decreased the levels of LDL and systolic BP.
	Lynn, 2014	47 healthy adults, aged 30–50 years	250 mL of tart cherry juice or control drink per day	6 weeks	A total of 250 mL of tart cherry juice (30 mL concentrate—Cherry Active) diluted in water or the same volume of control drink-lemonade (Sprite) was consumed per day.	Tart cherry juice had no effect on arterial stiffness, c-reactive protein, and risk markers for cardiovascular disease. Some antioxidant effects can be observed among healthy adults.
	Johnson, 2020	19 participants with metabolic syndrome, aged 20–60 years	480 mL of tart cherry juice or control drink per day	12 weeks	The tart cherry juice consisted of Montmorency tart cherry juice and concentrate (Indian Summer, Inc., Traverse City, MI, USA). The tart cherry juice and the placebo-control drink were consumed twice a day (240 mL each, in the morning and in the evening).	Tart cherry juice decreased the levels of LDL and total cholesterol. There were no significant effects on hemodynamic or arterial stiffness parameters.
**Bergamot**	Cicero, 2019	90 overweight participants with dyslipidaemia; aged 20–75 years	pill with bergamot extract (120-mg flavonoids/pill) or placebo pill; two pills per day	8 weeks	Before the treatment period, there was the diet standardization and physical activity period (2 weeks). A patient from the high-dose group was given two boxes with active treatment-Colber (from Esserre Pharma srl, Rome, Italy) containing bergamot extract (120 mg flavonoids/pill). A patient from the low-dose group was given one box with an active treatment and one with placebo. The patient from the placebo group was given two boxes with placebo pills. Each patient had 170 pills and took one tablet from each of two boxes at bedtime.	Improvement in TG, LDL; total cholesterol, C-reactive protein, and tumour necrosis factor-α decreased; improved lipid and glucose metabolism, adipokines pattern, and systemic inflammation in overweight subjects with dyslipidaemia.
	Ferro, 2020	102 patients with liver steatosis; aged 30–75 years	300 mg/day or placebo	12 weeks	Participants received a nutraceutical from Bergamot and wild cardoon or placebo.	Liver fat content reduction, especially in participants over 50 years.
**Pycnogenol**	Belcaro, 2013	130 participants with metabolic syndrome, aged 45–55 years	150 mg/day or placebo	6 months	The 50-mg tablets with Pycnogenol, from GEFA (Chateaugiron, France), were taken three times a day (total 150 mg of Pycnogenol per day)	Pycnogenol supplementation decreased levels of TG, fasting glucose, and plasma free radicals and increased the HDL cholesterol level. Pycnogenol decreased waist circumference and blood pressure.
	Enseleit, 2012	23 patients with coronary artery disease, aged 49–73 years	200 mg/day	8 weeks	-	Pycnogenol supplementation improved endothelial function in patients with coronary artery disease by reducing oxidative stress.
	Sedighiyan, 2018	38 obese women, aged 18–45 years	150 mg/day or placebo	2 months	Women received a weight loss diet and tablets (the PBE group) with 150 mg pine bark extract, from Source Naturals (CA, USA) and the placebo group received one placebo tablet (containing starch powder) per day for 2 months with a meal.	Pine bark extract had positive effects on TG, fasting blood sugar, HDL, and BP. It had a protective effect on metabolic syndrome in obesity.
**Beetroot juice**	Avoort, 2021	77 (pre)hypertensive adults, mean age 65 y	250–300 g of nitrate-rich vegetables consumption (containing ∼350–400 mg nitrate/∼6–6.5 mmol nitrate) vs. 70 mL BRJ drink (400 mg nitrate) vs. control (usual dietary behaviour)	12 weeks	Personalized dietary intervention vs. concentrated red beetroot juice (Beet-it Sport^®^, 70 mL; James White Drinks Ltd.) vs. usual diet.	A prolonged dietary intervention with high-nitrate vegetable intake lowered SBP in (pre)hypertensive middle-aged and older adults. DBP did not change in any of the groups.
	Gilchrist, 2013	27 adults with type 2 diabetes, mean age 67 y	250 mL BRJ (7.5 mmol of nitrate) daily or 250 mL nitrate-depleted BRJ (0.002 mmol of nitrate)	2 weeks	BRJ sold as a beverage. They used their own procedure for the production of placebo juice, using a column containing a nitrate-specific anion-exchange resin (Purolite A520e).	Supplementation with 7.5 mmol of nitrate per day did not lower BP or improve endothelial function in individuals with type 2 diabetes.
	Jones, 2019	20 healthy older adults, mean age 63 y	70 mL NO^3−^ rich BRJ (400 mg of NO^3−^) a day vs. placebo	28 days (±7)	BRJ drink (Beet-It Sport Shot 400). Prune juice was used for the placebo.	BRJ ingestion potentially improved BP. A decrease in SBP (−6 mmHg) and DBP (−4 mmHg) was significant (*p* = 0.04; *p* = 0.01, respectively). BP remained unchanged in the placebo group.
	Coles, 2012	30 healthy adults, mean age 42 y	500 g of beetroot and apple juice (BJ) containing 15 mmol nitrate/L or a placebo juice	24 h (measurements every hour after consumption)	Beetroot and apple juice (72% beetroots and 28% apples) vs. placebo (apple) juice; both supplied by Sunraysia Natural Beverage Company, Melbourne, VIC.	Trend of a lower SBP at 6 h after drinking BJ relative to placebo. Only men demonstrated a significant reduction in SBP (4–5 mmHg) at 6 h after BJ consumption.
	Velmurugan, 2016	67 adults with untreated hypercholesterolemia, mean age 53 y	250 mL nitrate-rich BRJ vs. placebo (nitrate-depleted BRJ)	6 weeks	For BRJ, they used James Whites Drinks. The placebo juice was generated from the same batch of nitrate-replete juice by using a standard anion exchange resin, as described in the protocol.	Dietary nitrate ingestion lowered SBP (−4 mmHg) and improved vascular function (increase in the FMD response with a worsening in the placebo group) in hypercholesterolemic patients.

**Table 2:** Supplement effectiveness review based on the included articles (*n* = 15).

Name of the Supplement	Author Year	Sample	Supplement Administration Protocol	Placebo Group	Duration	Form of Supplement	Findings
**Resveratrol**	Pollack, 2017	older glucose-intolerant adults aged 50–80 years (*n* = 30)	A resveratrol dose of 1500 mg twice daily was administered to the initial nine participants; however, because of gastrointestinal side effects, subsequent participants received 1000 mg twice daily. The study consisted of two randomly assigned 6-week treatment periods (resveratrol and placebo). Participants were instructed to begin the study drug on the evening following the baseline measurements and continue through the evening prior to metabolic testing at the end of each treatment period. Following a 3-week washout period, the participants crossed over to the other intervention for the second 6-week treatment period.	Older glucose-intolerant adults aged 50–80 years (*n* = 30)	6 weeks	Resveratrol capsules (RevGenetics Corporation)	No changes in glucose tolerance, insulin sensitivity, weight, blood pressure, or lipid profile; however, resveratrol treatment may have beneficial effects on vascular function
	Ligt, 2020	41 men and women (BMI: 27–35 kg/m^2^; aged 40–70 y	A total of 150 mg/d of resveratrol (*n* = 20) was administered in two doses of 75 mg per day—1 during lunch and 1 during dinner.	21 persons	6 months	Trans-resveratrol (resVida, 99.9%; provided by DSM Nutritional Products) capsules	No differences were found in intrahepatic lipid, body composition, blood pressure, energy metabolism, physical performance, or quality of life and sleep between treatment arms.
**Cherry juice**	Desai, 2018	11 healthy, recreationally active participants; 30 ± 10 years	260 mL of Montmorency tart cherry juice or placebo per day		20 days	A total of 30 mL of Montmorency tart cherry concentrate (Cherry Active, Active Edge Ltd., Hanworth, UK) and 100 mL of water gave 130 mL of tart cherry juice, and the placebo drink was made with 30 mL of commercially available fruit-flavoured cordial (Cherries and Berries, Morrisons Bradford, UK) and 100 mL of water. The amount of 130 mL of drinks was consumed twice a day (in the morning and in the evening).	Consumption of Montmorency tart cherry juice in addition to exercising did not give an improvement on cardio-metabolic biomarkers.
**Beetroot juice**	Asgary, 2016	24 hypertensive subjects aged 25–68 years old	250 mL/day raw beet juice (RBJ) vs. 250 g/day cooked beet (CB)	NA	2 weeks, followed by a 2-week washout period before entering the second phase of the study and receiving alternate treatment	Participants own preparation according to instruction	Both RBJ and CB were effective in improving BP, endothelial function, and systemic inflammation. BRJ had greater antihypertensive effects. More improvement was observed in endothelial function and systemic inflammation with RBJ compared with CB.
	Avoort, 2020	30 recreationally active individuals, mean age 24 years	250 g vegetables (ca.400 mg nitrate daily) at lunch vs. 70 mL of red beetroot juice/day	NA	BP measurement fasting and 2.5 h after lunch on day 1, 4, and 7.	Participant own preparation vs. BRJ drink (Beet-it Sport; James White Drinks Ltd.).	Fasting mean systolic and diastolic BP did not change, but the mean systolic and diastolic BP assessed 2.5 h after lunch were significantly reduced in both groups (SBP −5.1 mm Hg and DBP −5.3 mm Hg).
	Bondonno, 2015	27 treated hypertensive individuals	2 × 70 mL concentrated nitrate-rich beetroot juice (70 mL with breakfast and 70 mL with dinner) vs. placebo	2 × 70 mL of nitrate-depleted beetroot juice (70 mL with breakfast and 70 mL with dinner)	1 week	Concentrated nitrate-rich beetroot-juice beverage (Beet it; James White Drinks Ltd.) vs. nitrate-depleted beetroot juice beverage (Beet it; James White Drinks Ltd.).	No differences in home BP and 24-h ambulatory BP were observed with the 1-wk intake of nitrate-rich beetroot juice in comparison with the placebo.
	Gallardo, 2021	9 healthy older subjects, mean age 70 y	3.3 mL/kg of concentrated beetroot juice containing 0, 200, or 400 µmol/kg of nitrate. Pts received all doses, with a 1- to 2-week washout period between visits.	Placebo: 3.3 mL/kg BRJ devoid of NO^3−^(0)	2 h	A commercial concentrated beetroot juice (BRJ) supplement (Beet It Sport, James White Drinks, Ipswich, UK); placebo was produced by extracting NO^3−^ from BRJ using a highly selective anion exchange resin (i.e., Purolite a520e).	No significant changes were found in systolic, diastolic, or mean arterial blood pressure.
	Hobbs, 2012	18 normotensive individuals	Study 1. (*n* = 18) one intervention: 0 g BJ (500 g water) as a control; or 100 g BJ with 400 g water; or 250 g BJ with 250 g water or 500 g BRJStudy 2. (*n* = 14) one intervention: 200 g white bread (control, 0 g beetroot enrichment); or 200 g bread enriched with either white or red beetroot (comprising 50% of the total weight of dough before baking).	NA	24 h	BRJ—James White Drinks Limited. The control drink and BJ drinks were all diluted with low-nitrate mineral water (The Buxton Mineral Water Company Limited). Bread products (Eccentricities Limited).	BJ consumption significantly, and in a near dose-dependent manner, lowered SBP and DBP over a period of 24 h, compared with the water control. Bread products enriched with 100 g of red or white beetroot lowered SBP and DBP over a period of 24 h, with no statistical differences between the varieties.
	Avoort, 2020	15 healthy men and women, mean age 24 y	400 mg of nitrate at lunch, provided through nitrate-rich vegetables and dietary counselling, or 70 mL beetroot juice supplementation.	NA	1 week, with 1-week washout	Dietary instruction for patients; (70 mL) of red beetroot juice (Beet-it Sport; James White Drinks Ltd.)	Fasting mean SBP and DBP did not change, but mean SBP and DBP assessed 2.5 h after lunch were significantly reduced throughout both intervention periods (*p* < 0.05), with no differences between beetroot juice and nitrate-rich vegetables.
	Jakubcik, 2021	11 healthy adults, 18–50 years old	250 mL of BR containing one of the following: (i) high NO^3−^, low NO^2−^ (HL; 572 mg NO^3−^, 32 mg NO^2−^); (ii) medium NO^3−^, medium NO^2−^ (MM; 280 mg NO^3−^, 237 mg NO^2−^); (iii) low NO^3−^, medium NO^2−^ (LM; 43 mg NO^3−^, 262 mg NO^2−^); (iv) placebo (PL; low NO^3−^, low NO^2−^: 8 mg NO^3−^, 5.8 mg NO^2−^).	placebo (PL; low NO^3−^, low NO^2−^: 8 mg NO^3−^, 5.8 mg NO^2−^)	6 h	Prepared according to the protocol	There was no effect of NO^3−^ or NO^2−^ ingestion on SBP or DBP compared to PL. Mean arterial pressure decreased following the consumption of HL, MM, and LM; however, in comparison, LM showed its effect 2 h later.
	Kapil, 2015	68 hypertensive patients, 18–85 years old	250 mL BRJ vs. 250 mL placebo	*n* = 34	4 weeks	250 mL of beetroot juice, James White Drinks Ltd., Ipswich, UK; placebo 250 mL nitrate-depleted beetroot juice, James White Drinks Ltd., Ipswich, UK.	Mean reduction in clinic BP was 7.7/2.4 mmHg; 24 h ambulatory blood pressure was reduced by 7.7/5.2 mmHg, and home blood pressure was reduced by 8.1/3.8 mmHg. Endothelial function improved by ~20%, and arterial stiffness reduced by 0.59 m/s after dietary nitrate consumption with no change after placebo
	McDonagh, 2018	10 healthy, normotensive males, age 24 y	55 mL of a concentrated beetroot juice drink (∼5.76 mmol) (BR); 456 mL of a non-concentrated beetroot juice drink (BL), and a solid beetroot flapjack (BF; 60 g). A drink containing soluble beetroot crystals (BC; ∼1.40 mmol NO^3−^) and a control drink (CON; 70 mL deionised water) were also ingested.	NA	24 h	Concentrated beetroot drink (55 mL of Beet It Sport Stamina Shot, James White Drinks, Ltd., Ipswich, UK), a non-concentrated beetroot drink (456 mL of Beet It Organic Beetroot Juice, James White Drinks, Ltd., Ipswich, UK) and a beetroot flapjack (60 g of Beet It Pro Elite Sport Flapjack, James White Drinks, Ltd., Ipswich, UK). In addition, subjects consumed the recommended dose (5 g dissolved in 114 mL of water; 1.40 mmol NO^3−^ and ∼0.07 mmol NO^2−^ ) of Concentrated Organic Beetroot Crystals (SuperBeets Canister; Neogenis, now known as HumanN, Texas, US), and a control drink (70 mL deionised water) that contained negligible NO^3−^ and NO^2−^ contents.	BR, but not BF, BL, and BC, reduced systolic (∼5 mmHg) and mean arterial pressure (∼3–4 mmHg; *p* < 0.05), whereas BF reduced diastolic BP (∼4 mmHg; *p* < 0.05).
	Siervo, 2020	47 middle-aged and older participants, age: 50–70 y, with elevated blood pressure	(1) high-nitrate beetroot juice (∼400 mg nitrate) and folic acid (∼5 mg folic acid) (N + F), (2) high-nitrate beetroot juice and placebo (N + P), or (3) nitrate-depleted beetroot juice and placebo (P + P).	*n* = 15	60 days	N + F: 70 mL of concentrated beetroot juice (Beet It shots, James White Ltd.) and 1 capsule of folate (folic acid, 5 mg, Bio-Tech Pharmacal Inc.). N + P: 70 mL of concentrated beetroot juice as group 1 (Beet It, James White Ltd.) and 1 placebo capsule containing sucrose powder. P + P: 70 mL of nitrate-depleted beetroot juice (<1 mg inorganic nitrate, James White Ltd.) and a placebo capsule containing sucrose powder.	After 60 d, 24-h systolic BP dropped by −10.8 ± 9.8 mm Hg (*p* < 0.001), −6.1 ± 13.2 mm Hg (*p* = 0.03), and −0.3 ± 9.7 mm Hg (*p* = 0.83) in the N + P, N + F, and P + P groups, respectively. There was a significant decrease in 24-h diastolic BP in the N + P group (−5.4 ± 5.0 mm Hg, *p* = 0.004), whereas changes were not significant in the N + F (−1.8 ± 8.1 mm Hg, *p* = 0.32) and P + P (1.6 ± 8.3 mm Hg, *p* = 0.43) groups.
	Stanaway, 2019	13 younger (18–30 years) and 11 older (50–70 years) recreationally active adults	Pts consumed either 150 mL of nitrate-rich beetroot juice (BR; 10.5 mmol nitrate) or placebo (PL; 1 mmol nitrate).	The same as a study group	BP measurements before and 145 min after supplementation	Beetroot juice, extracted from fresh washed beetroots (beta vulgaris ‘Pablo’), was blended with other fruit juices to provide a standardized beetroot juice drink with a constant soluble solid concentration and nitrate concentration (10.5 mmol/150 mL). The placebo beetroot drink was produced using beetroot juice concentrate and standardized to the same constant soluble solids concentration but contained a low nitrate concentration (1 mmol/150 mL).	BR consumption significantly increased plasma nitrate and nitrite concentrations and reduced SBP (*p* < 0.001) in both age groups and reduced DBP (*p* = 0.013) in older adults.
**Barberry**	Lazavi, 2018	46 diabetic patients; aged 30–70 years	200 mL of barberry juice daily	Control group received no intervention	8 weeks	The concentrated barberry juice was diluted with water.	The consumption of barberry juice decreased systolic and diastolic blood pressure, TG, fasting blood sugar, and total cholesterol. The risk of cardiovascular diseases might decrease in patients with diabetes.

## Supplementary Materials

The following are available online at https://www.mdpi.com/article/10.3390/antiox11081419/s1, Table S1. Search terms used for database searches, based on the PICO System for search strategy development.
