# Supplementary material for: The Effect of Herbal Supplements on Blood Pressure: Systematic Review and Meta-Analysis
Source: Antioxidants (Basel). 2022 Jul 22;11(8):1419. doi: 10.3390/antiox11081419 (PMC9332300; doi:10.3390/antiox11081419)
Supplement: Supplementary file 1 [file antioxidants-11-01419-s001.zip › antioxidants-1788924-supplementary.pdf]

Table S1. Search terms used for database searches, based on the PICO System for search strategy development.

Compound	Population	Outcome
Beetroot juice	Healthy adults…	cardiovascular disease
Cherry juice		blood pressure
Resveratrol		hypertension (except pulmonary)
Barberry		endothelial function (additional
Bergamot		indicators, such as flow-mediated
Cherry juice		dilation, FMD; pulse wave
Pycnogenol		velocity, PVW)
		vascular function
		arterial stiffness
